# A response to: *BAK1* gene variation and abdominal aortic aneurysms—results may have been prematurely overrated. Questions of sequence fidelity, intraorganismal genetic heterogeneity, the nature of pseudogenes, and RNA editing

**DOI:** 10.1002/humu.21323

**Published:** 2010-09-28

**Authors:** Bruce Gottlieb, Lorraine E Chalifour, Morris Schweitzer

**Affiliations:** 1 Lady Davis Institute for Medical Research, Jewish General HospitalMontréal, Québec, Canada; 2 Department of Human Genetics, McGill UniversityMontréal, Québec, Canada; 3 Division of Experimental Medicine, Department of Medicine, McGill UniversityMontréal, Québec, Canada; 4 Department of Endocrinology, Jewish General HospitalMontréal, Québec, Canada

We thank Küry et al. [2010] for their letter. They raise a number of issues with regard to our initial article that reported on variations in BAK1 gene (MIM# 600516) sequence expressed in the abdominal aorta (AA) tissue when compared with leukocyte genomic DNA sequence [Gottlieb et al., 2009]. In a subsequent letter [Gottlieb et al., 2010], we noted that when we compared the genomic *BAK1* sequence in matching blood and tissue samples, the blood and tissue *BAK1* genomic sequences were identical to the reference *BAK1* genomic sequence, which primarily suggested that an unusual form of RNA editing could possible be involved.

Küry et al. [2010] have raised three issues with regard to possible explanations for our results.

1. That the *BAK1* sequence variants are due to somatic events, which they dismiss as highly unlikely. We agree with them that this is unlikely, for the simple reason that the genomic DNA appears to be similar, which is the reason that we did not comment on this earlier.
2. That differences between DNA and RNA sequences must lie at the RNA level, which they dismiss as impossible because the form of RNA editing that we observed except for C>T (c.454C>T) had not been reported. However, both G>A (c.336G>A) and T>C (c.366T>C) have now been reported in the TPH2 gene isolated from human amygdala samples [Grohmann et al., 2010]. Although the exact mechanism has yet to be elucidated it seems somewhat premature to dismiss the validity of this being due to RNA editing just because no possible mechanism has yet been discovered.
3. They again raise the possibility that that the sequence variants that we report are due to a Bcl-2-like 7 pseudogene 1, alias BAK2 with gene ID number 600, NC_000020.10, the so-called *BAK2* pseudogene, being sequenced. We have already described in detail the techniques and safeguards that we have used to ensure that *BAK2* was not sequenced in our response [Gottlieb et al., 2010] to a previous letter [Hatchwell, 2010].

It is clear that the *BAK1* sequence we amplified from the cDNA prepared from our DNAase treated RNA (not, of course, RNAase treated, as mistakenly noted by Küry et al. [2010]) resembles both *BAK1* and *BAK2* yet copies neither exactly. At amino acid 2 the chromosome 20 *BAK2* reference sequence of NC_000020.10 has the sequence ATG/GCC/TCG. In contrast, *BAK1* mRNA (NM_001188), and all our samples isolated from AAA and all AA cDNA samples have the sequence ATG/GCT/TCG (Table 1). In Figure 2, Küry et al. [2010] reference five other *BAK2* sequences to indicate that there is heterogeneity at nucleotide 217. The results, however, are likely from only three different isolates not five. *BAK2* chromosome 20 sequences NC_000020.10 and NT_011362 contain ATG/GCC/TCG, with both sequences having the same citation [Deloukas et al., 2001], suggesting that they are from the same isolate. *BAK2* chromosome 20 sequence NG_000850.5 has ATG/GCT/TCG and is referenced in Kiefer et al. [1995]. This publication was the original description of the cloning and sequencing of *BAK1*, *BAK2*, and *BAK3*. Here these authors categorize *BAK3* as a pseudogene, but indicate that *BAK2* is not a processed gene. The chromosome 20 *BAK2* sequences of AC_000152.1 and NW_00183664 contain ATG/GCT/TCG from the DNA of Dr. J. Craig Venter [Levy et al., 2007] and so are also from a single isolate. It is interesting to note that Venter's DNA sequence appears to be somewhat of an outlier when compared to the other complete human genomes so far sequenced [Ahn et al., 2009]. Please note that we compared our results to the reference and not ancillary sequences, and that regardless of the sequence variations among the BAK2 genes it is clear that the sequence we amplified contains the sequence present in *BAK1*.

**Table 1:** Sequence of *BAK1* and *BAK2* cDNA

*BAK1* Amino Acid #	2	145
*BAK1* Nucleotide #	217	645
*BAK1* reference NM_001188	ATG/GC**T**/TCG	CCA/GC**A**/TGG
AAA sample	ATG/GC**T**/TCG	CCA/GC**A**/TGG
AA cDNA	ATG/GC**T**/TCG	CCA/GC**A**/TGG
*BAK2* reference NC_000020.10	ATG/GCC/TCG	CCA/GCG/TGG
*BAK2* NT_011362^a^	ATG/GCC/TCG	CCA/GCG/TGG
*BAK2* NG_000850.5	ATG/GCT/TCG	CCA/GCG/TGG
*BAK2* AC_00152.1 and NW_00183664^b^	ATG/GCT/TCG	CCA/GCG/TGG

a This sequence has the same citation as the reference sequence NC_000020.10 [Deloukas et al., 2001].

b Both of these sequences are from a single isolate [Levy et al., 2007].

In Figure 2, Küry et al. [2010] also compared the sequencing results for the same *BAK2* sequences and *BAK1* sequence at nucleotide 645. All the *BAK2* sequences have the CCA/GCG/TGG sequence, whereas the *BAK1* sequence is CCA/GCA/TGG. All of our samples are the same as the *BAK1* sequence and contain the sequence CCA/GCA/TGG (Table 1).

We believe that our critics have been very subjective in their use of sequence data in that they chose not to discuss the concordance of our results at nucleotides 217 and 645 with the *BAK1* sequence, but only to discuss the discordance at 217 in the *BAK2* sequences by relying on three variant *BAK2* sequences. This would appear to be a good example of a selective use of sequence data, so that even though we reported on actual sequence data from individuals, they chose to solely rely on reported data from these three cases.

Although we feel that we are clearly dealing with single nucleotide polymorphisms (SNPs) in *BAK1*, as noted previously in the original article reporting *BAK2* in chromosome 20 [Keiffer et al., 1995], the authors reported that it could not be considered a processed gene. Finally, a recent study has identified correlation of 34 trait-associated SNPs with copy number variations, and interestingly, one of the genes identified was *BAK1* [Conrad et al. 2010]. Although the associated trait was testicular germ cell tumors, it suggests that *BAK1* SNPs have the potential to be disease associated.

Again, we are grateful to Küry et al. [2010] for raising a number of issues that are likely to be of importance as we uncover increasing amounts of human genome variation. In particular, their letter raises a number of key questions that are only likely to increase in significance in the coming years. These questions include the following:

1. What is the degree and significance of gene sequence fidelity? Clearly, the authors of this letter have placed a great deal of faith in the importance of gene fidelity, as they have selectively used this principle to very rigidly separate *BAK1* from *BAK2* sequence data.
2. What is the importance of intraorganismal genetic heterogeneity? Differences between diseased, nondiseased tissues, and blood have long been a staple of cancer research. Do they also occur in other diseases? What are the possible effects of intraorganismal genetic heterogeneity on the controversies surrounding GWAS?
3. What actually are pseudogenes, how are they related to similar regular genes and how does the discovery of some pseudogenes being expressed [Brunson et al., 2010], affect our ideas about their genetic significance? Can pseudogenes indeed play a role in genetics?
4. What forms of RNA editing are possible and what might be their significance, particularly in establishing genotype to phenotype relationships?

Finally, The question of prematurely overrating results is clearly a fact of life in modern science. As Küry et al. [2010] noted, it was not us that did so in our article, but rather the popular Scientific Press. The fact that such behavior goes on constantly is perhaps a measure of the importance of public relations (PR) in modern scientific research. All large universities and research institutions now have a PR department, whose goal is to make certain that their research reputation is suitably promoted and enhanced. This has lead to a veritable orgy of press releases announcing countless major scientific breakthroughs, most of which in the harsh light of reality tend to be highly overrated. Further, such behavior is not just limited to the PR departments of institutions, but also the PR departments of some of our most highly rated scientific publications. Perhaps it is time to seriously consider the value of such press releases, as they are in many ways resulting in irresponsible and even poor science. This can perhaps best be illustrated by the numerous press conferences that have announced the results of experiments that have not yet been completed or even undertaken! As scientist and researchers we are constantly being reminded of the positive benefits of publicity, but perhaps it is also time to consider the negative aspects as well.
